# Synergistic effect of proton irradiation and strain on the mechanical properties of polyimide fibers

**DOI:** 10.1039/d0ra07039d

**Published:** 2020-10-29

**Authors:** Ju Dandan, Sun Chengyue, Wang Hao, Wang Xinmin, Wu Yiyong, Dong Zhixin, Qiu Xuepeng

**Affiliations:** Harbin Institute of Technology Harbin 150001 China wuyiyong@hit.edu.cn; Changchun Institute of Applied Chemistry, Chinese Academy of Sciences Changchun 130022 China xp_q@ciac.ac.cn

## Abstract

The damage behaviors of polyimide fiber after 150 keV proton irradiation and the synergistic effect of proton irradiation and strain were investigated. Changes in the mechanical properties, free radicals, element content, and element chemical state of the polyimide fiber before and after 150 keV proton irradiation were investigated. The results showed that the tensile strength and elongation at break of the material decreased significantly after proton irradiation. The synergistic effect of proton irradiation and strain weakened the reduction of mechanical properties caused by single proton irradiation. After proton irradiation and the combination of proton irradiation and strain, pyrolytic carbon free radicals were generated. According to XPS analysis, the proton-irradiated polyimide fiber underwent complex denitrification and deoxygenation reactions, and carbon enrichment appeared on the surface of the material.

## Abstract 2

Compared with the single irradiated sample, the tensile strength and elongation at break after the combination of proton irradiation and strain increased, indicating that introducing strain during irradiation will weaken the damage of irradiation.

## Graphical abstract

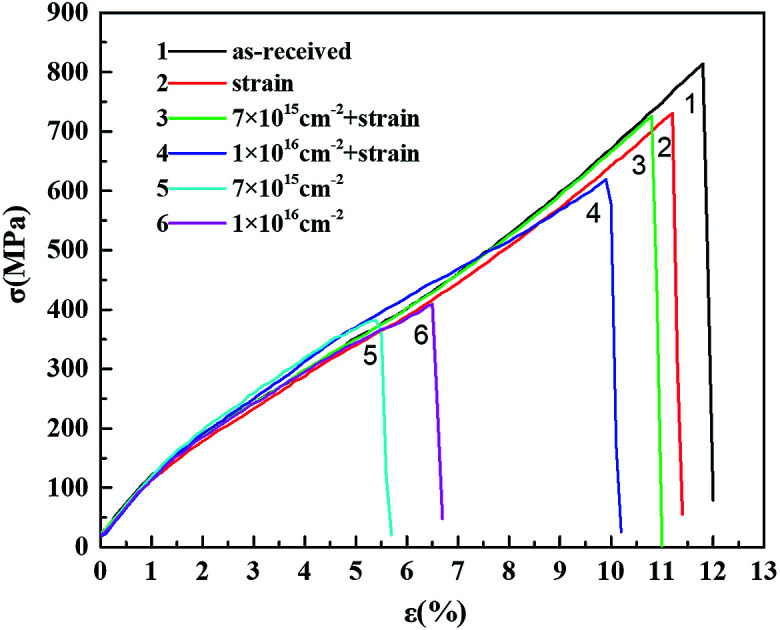

## 1. Introduction

Polyimide fiber has high strength and modulus and high radiation resistance,^1^ which enable it to be used as lightweight cable jackets for spacecraft and rockets as well as in fiber-reinforced composites for space applications. As materials used in space may be subjected to substantial quantities of high-energy radiation, it is important that the response of the polyimide fiber to high-energy radiation should be evaluated.

A large number of space experiments with polyimide films have been implemented over a period of several years. The radiation sensitivity of Kapton to 3 MeV proton irradiation was investigated and the results revealed that the elongation to break, break stress, and the fracture energy of polymers decreased significantly on radiolysis. Moreover, the elongation at break was similar in magnitude to that induced by 2 MeV electron irradiation with the same dosage.^2^ The electron, proton, or both combined irradiations all induced bond break and cross-linking of polyimide molecules, while proton irradiation could break the PI bond easier than electron irradiation and then led to the formation of a graphite-like structure at the surface area of the samples.^3^ Proton irradiation increased the initial friction coefficient and decreased the steady friction coefficient of polyimide.^4^ The wear rate of the irradiated PI decreased in the order: electron irradiation > proton irradiation > combined irradiation.^5^ The proton irradiation could also control the refractive index of the polyimide. The refractive index of the fluorinated polyimide increased by about 0.21%, which was comparable to the value (0.35%) of SiO_2_ glass when they were irradiated by protons at a similar fluence. This result provided to be a good method for fabricating a high-performance polymer-based optical waveguide.^6^ After proton irradiation, the surface roughness of PI increased apparently. The enrichment of carbon atom on the surface due to the bond breaking and reconstruction during the irradiation resulted in the degradation of the optical properties.^7^ The electron paramagnetic resonance (EPR) results indicated that the proton irradiation induced the formation of pyrolytic carbon free-radicals with a *g* value of 2.0025, and the population of free radicals increased with the irradiation fluence. During the post storage after irradiation, the free-radical population decreased following the sum of exponential and linear mode with the storage time. In the meantime, the recovery of the degraded optical absorbance of the polyimide followed a similar mode to that of free radicals.^8–10^ Compared with the case of polyimide, the free radical population of a SiO_2_/PI composite was much higher and increased faster with the irradiation fluence and the nano-SiO_2_ film thickness.^11^

However for the polyimide fiber, there were significant differences in the performance due to the high degree of crystallization or orientation.^12–14^ Wu Dezhen conducted a detailed study on the performance changes of high-strength and high-modulus polyimide fibers in high temperature, strong acid, strong alkali, and ultraviolet irradiation environments, and compared them with P84. After 150 h UV radiation, the mechanical properties of the fiber remained above 90%, which was better than the resistance of P84.^15^ Zhao Yong investigated the atomic oxygen resistance of a phosphorus-containing PI fiber. The results indicated that the phosphorus-containing PI fibers exhibited a denser surface morphology and lower weight loss compared with that of the pure PI fibers.^16,17^

However, under the irradiation of space-charged particles, the property evolution of the high-performance polyimide fiber and the corresponding damage mechanism have not been reported. At the same time, the polyimide fiber is usually subjected to stress/strain in the flexible mechanism applications in space. This study investigated the effect of proton irradiation on the mechanical properties, free radical evolution, and element content change of the polyimide fiber. On the other hand, the synergistic effect of proton irradiation and strain was also researched to simulate the environmental conditions in practical applications.^18^ This research could provide a certain experimental basis for the application of polyimide fiber in space.

## 2. Experiment

### 2.1 Materials

**Fig. 1:**
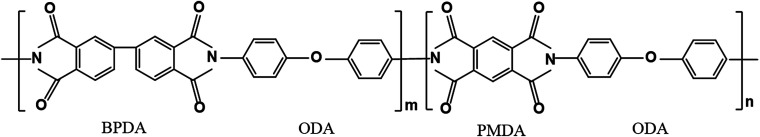
Molecular structure of the polyimide fiber.

In this study, the polyimide fiber was kindly supplied by the Changchun Institute of Applied Chemistry, Chinese Academy of Sciences. The diameter of the fiber was 16 ± 1.7 μm. For the convenience of testing and characterization, the long fiber was cut into 50 mm lengths. The molecular structure is shown in Fig. 1. Its synthetic monomers comprised: BPDA (3,3,4,4-biphenyl dianhydride), PMDA (1,2,4,5-pyromellitic dianhydride), and ODA (4,4′-diaminodiphenyl ether).

### 2.2 Test equipment and irradiation parameters

In this study, the selected proton irradiation energy was 150 keV. The equipment included a 
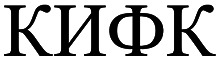
 integrated irradiation simulator and space positive ion accelerator at the National Key Laboratory of Space Environment Materials Behavior and Evaluation Technology of Harbin Institute of Technology. The specific irradiation test parameters are shown in Table 1.

**Table 1:** Irradiation test parameters of the polyimide fiber

Particle type	Energy	Environment	Flux (cm^−2^ s^−1^)	Fluence (cm^−2^)
Proton	150 keV	Vacuum	1.25 × 10^12^	2 × 10^15^
				4 × 10^15^
				7 × 10^15^
				1 × 10^16^
				2 × 10^16^
Proton and strain	150 keV	Vacuum	1.25 × 10^12^	7 × 10^15^
				1 × 10^16^

**Fig. 2:**
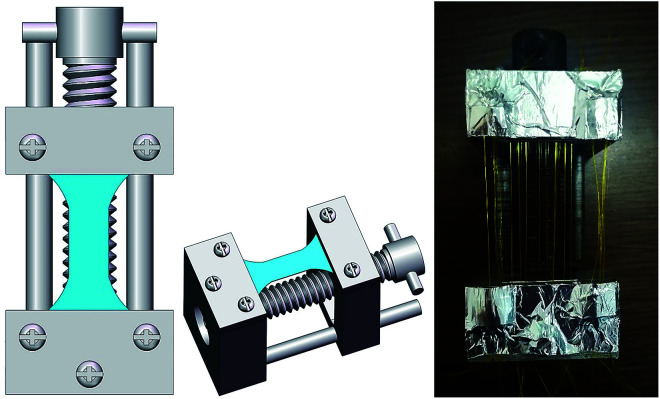
Schematic diagram (left, middle) and photo (right) of the fixture with constant strain.

**Fig. 3:**
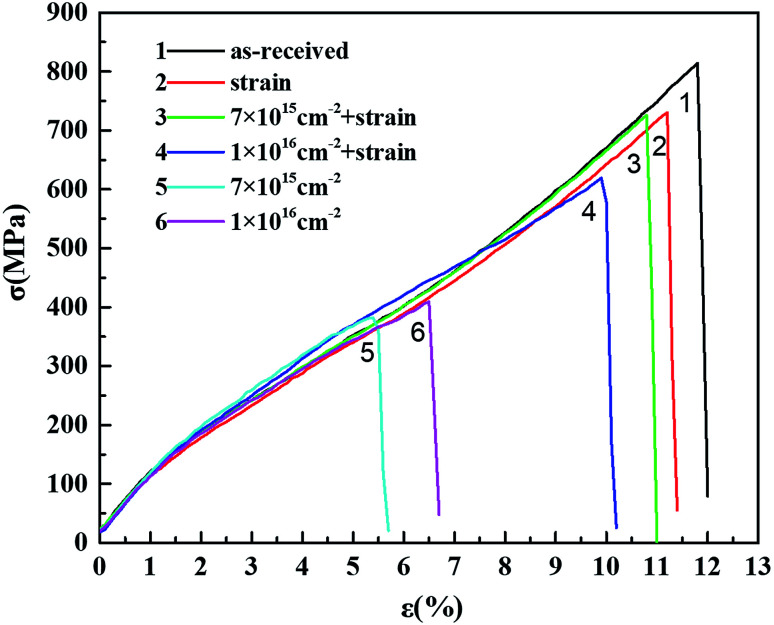
Tensile stess–strain curves of the polyimide fiber before and after the combination of proton irradiation and strain.

In order to study the synergistic effect of proton irradiation and strain on the mechanical properties of the fibers, we carried out a combination of proton irradiation and strain experiments by selecting two fluences of 7 × 10^15^ cm^−2^ and 1 × 10^16^ cm^−2^ under the same experimental conditions as 150 keV proton irradiation. This experiment was realized by a self-made fixture with constant strain, of which the schematic diagram and physical diagram are shown in Fig. 2. The sample was clamped by the clip and fixed by screws. The lower module could be kept in a stationary state, while the upper module could be raised under the action of the middle lead screw, so the sample could be elongated following the upper module. The strain of the sample could be calculated from the original and the final distance of the two modules. Meanwhile, the constant strain of the sample could be ensured by the self-locking function of the lead screw, which does not slide downward during the upward movement and after stopping the movement. According to the stress–strain curves of the polyimide fiber before and after proton irradiation (shown in Fig. 3), the strain applied to the sample was determined to be 5% ± 0.5%. At the same time, in order to eliminate the influence of a single strain factor on the mechanical properties of the polyimide fiber, a test with only 5% ± 0.5% strain and no irradiation was prepared as the control under the same vacuum conditions with 4 h.

### 2.3 Tensile measurement

The tensile tests before and after irradiation were carried out in accordance with the ASTM D3379-75 standard, which involved using an XQ-1 fiber strength meter at a tensile rate of 20 mm min^−1^. Here, 10 to 20 fiber monofilaments were tested for each sample to obtain an average value.

### 2.4 Electron paramagnetic resonance analysis

The electronic paramagnetic resonance analysis was carried out with A200 electronic paramagnetic resonance spectrometer equipment produced by Bruker, Germany. A Q-band microwave source was used in this experiment. The detailed parameters used were a center magnetic field of 3514.00 G with a sweep width of 70.00 G, microwave frequency of 100 kHz, time constant of 40.96 s, spectral line gain of 5.02 × 10^4^, and modulation amplitude of 1 G.

### 2.5 X-ray photoelectron spectroscopy

The surface element composition and chemical valence state were analyzed on a PHI5300 system X-ray photoelectron spectrometer manufactured by PHI Corporation of the United States. The X-rays were generated by MgKα (1253.6 eV) radiation, and the test power was 250 W and the voltage was 13 kV. The electron binding energies of all the peaks were corrected by the binding energy of the C 1s peak of 284.6 eV. The results were fitted by the Gauss–Lorentz function.

## 3. Results and discussion

### 3.1 Mechanical properties of the polyimide fiber before and after irradiation

#### 3.1.1 150 keV proton irradiation

The tensile properties of the polyimide fiber after proton irradiation are shown in Table 2. Compared with the non-irradiated sample, the tensile strength and elongation at break of the fiber after 150 keV proton irradiation declined significantly, and the degree increased with increasing the irradiation fluence. Shen Zicai *et al.* also studied the proton-irradiated polyimide film and showed that the tensile strength and elongation at break of the film decreased exponentially with the increase in the proton irradiation fluence.^19,20^ In this test, the tensile strength decreased from 0.79 GPa for the non-irradiated samples to 0.42 GPa, which was 46.8% lower. The elongation at break of the sample decreased from 11.82% to 6.60%, representing a decrease of 44.2%. However, the modulus of the sample only decreased slightly. For the PI films, under similar conditions, the tensile strength and elongation at break dropped by 25%.^21^ This might be due to the higher specific surface area of the fiber, which generated more free radicals; thus resulting in more damage, and therefore reducing the tensile strength and elongation at break.

**Table 2:** Tensile properties of the polyimide fiber before and after 150 keV proton irradiation

Fluence (cm^−2^)	Tensile strength (GPa)	Modulus (GPa)	Elongation at break (%)
0	0.79 ± 0.01	9.80 ± 0.10	11.82 ± 0.14
2 × 10^15^	0.47 ± 0.01	9.61 ± 0.11	7.33 ± 0.14
4 × 10^15^	0.41 ± 0.01	9.04 ± 0.14	6.26 ± 0.21
7 × 10^15^	0.38 ± 0.01	9.31 ± 0.10	5.85 ± 0.17
1 × 10^16^	0.42 ± 0.01	9.56 ± 0.09	6.60 ± 0.08
*F*	358.09	7.35	234.68
*P*	0	3.51 × 10^−5^	0

In order to confirm that these changes were caused by the effects of irradiation rather than random uncontrollable factors, the variance analysis (ANOVA) was used to evaluate the confidence level of the 150 keV proton irradiation effect on the mechanical properties of the polyimide fibers.

One-way ANOVA is a data processing method to judge whether the influence of a certain factor on the test index is significant. By calculating the ratio of the variance *S*_E_^2^, which reflects the data fluctuation caused by the test itself, and the variance *S*_A_^2^, which reflects the fluctuations caused by the investigated factor A, *F*_A_ can be obtained as per eqn (1). *F*_A_ represents the ratio of the variation between the groups and the variation within the group, and is used to determine whether the level of the A factor has an influence on the observed value. Actually, it can be used to compare the differences between the mean squares within the group and the mean squares between groups. The probability *P*, which reflects the factor A, has no influence on the data fluctuation and can be acquired by further calculation. By comparing the *P*-value with the significance index *α*, one can determine whether the factor A has an influence on the fluctuation of the experimental data.1
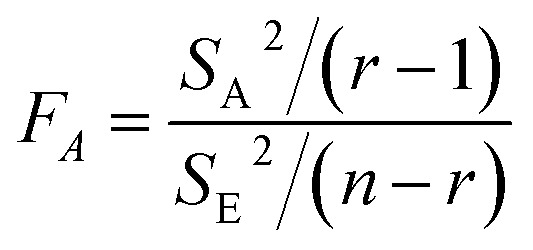


When analyzing the tensile property data obtained in this test, the factor A is the irradiation fluence, and the *P*-value indicates the probability that the null hypothesis that the irradiation has no effect on the tensile properties is true. Selecting *α* = 0.05 as the significance level, when *P* is less than *α*, the null hypothesis is rejected with a probability greater than (1 − *α*) 100%; that is to say, the confidence level of the effect of irradiation on the tensile properties is >95%. Thus, the confidence of the effect of irradiation on the different tensile properties of the polyimide fiber can be derived from the *P*-value.

The *F* statistics and *P*-values of the mechanical properties of the polyimide fiber before and after 150 keV proton irradiation are shown in Table 2. The results showed that the *P*-values of the tensile strength, modulus, and elongation at break of the sample were far less than 0.05, indicating that the proton irradiation had a significant impact on the mechanical properties of the polyimide fiber. Moreover, the *P*-values of tensile strength and elongation at break were both 0, indicating that the effects of proton irradiation on the tensile strength and elongation at break were much more significant than the modulus. Therefore, proton irradiation had an effect on the tensile properties of polyimide fibers, and as the irradiation fluence increased, the tensile strength, modulus, and elongation at break were all reduced.

#### 3.1.2 Synergistic effect of proton irradiation and strain

**Fig. 4:**
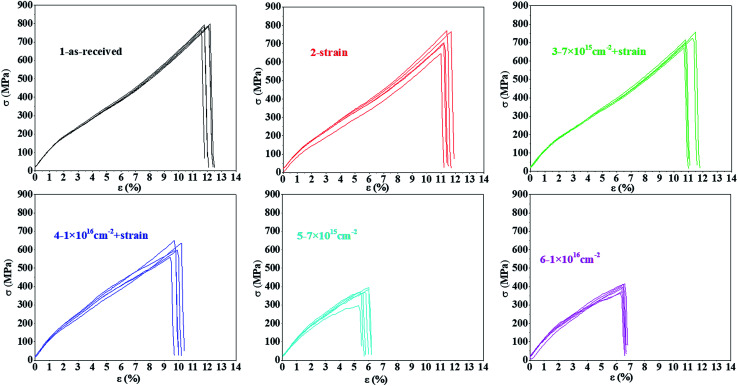
The five times repeated tensile stress–strain curves for each sample in Fig. 3.

Fig. 3 shows the tensile stress–strain curves for the original polyimide fiber with a single applied strain, the combination of proton irradiation and strain, and single irradiation, respectively. It can be seen from the table that compared with the original sample, a single application of 5% strain caused a certain decrease in the tensile strength and elongation at break, which were further reduced after the combination of proton irradiation and strain, indicating that the tensile properties damage of the sample after the combination of proton irradiation and strain were not simply caused by strain. On the other hand, compared with the single irradiated sample, the tensile strength and elongation at break of the sample after the combination of proton irradiation and strain were increased, indicating that introducing strain during irradiation will weaken the damage from the irradiation to the fiber. The five times repeated tensile stress–strain curves for each sample in Fig. 3 are shown in Fig. 4. Every curve of each sample illustrated a similar variation of tensile strength and elongation at break with proton irradiation and strain.

The statistical results are more clearly shown by the tensile parameter changes listed in Table 3. For comparison, the mechanical properties of certain samples after single 150 keV proton irradiation in Table 2 were repeated here. It can be seen from the table that compared with the tensile strength of 0.79 GPa of the original sample, it was decreased to 0.38 and 0.42 GPa after irradiation of 7 × 10^15^ cm^−2^ and 1 × 10^16^ cm^−2^, respectively. While, after strain was applied, the tensile strength was only reduced to 0.72 and 0.63 GPa, respectively. Similarly, after single irradiation, the elongation at break was decreased from 11.82% to 5.85% and 6.60%, respectively. After the combination of proton irradiation and strain, the elongation at break only decreased to 11.50% and 9.98%, respectively. The same study for polyimide films also showed a similar phenomenon.

**Table 3:** Tensile properties of the polyimide fiber before and after the combination of proton irradiation and strain

Fluence (cm^−2^)	Tensile strength (GPa)	Modulus (GPa)	Elongation at break (%)
0	0.79 ± 0.01	9.80 ± 0.10	11.82 ± 0.14
5% strain	0.74 ± 0.01	9.36 ± 0.05	11.33 ± 0.17
7 × 10^15^ cm^−2^	0.38 ± 0.01	9.31 ± 0.10	5.85 ± 0.17
7 × 10^15^ cm^−2^ + 5% strain	0.72 ± 0.02	9.42 ± 0.23	11.50 ± 0.17
1 × 10^16^ cm^−2^	0.42 ± 0.01	9.56 ± 0.09	6.60 ± 0.08
1 × 10^16^ cm^−2^ + 5% strain	0.63 ± 0.01	10.18 ± 0.09	9.98 ± 0.15

For the polyimide films, the combination of proton irradiation and strain increased the tensile strength while it reduced the elongation at break.^21^ This matter could be explained as follows. On the one hand, in the process of the combination of proton irradiation and strain, strain increased the free volume of the molecules, which accelerated the recovery of the free radical; therefore resulting in less damage than from the single irradiation. On the other hand, the irradiation-destroyed molecular chains could recombine in the direction of stretching, which enhanced the mechanical properties of the sample due to the orientation. Because the fiber is usually subjected to stress/strain in space applications, the study of the synergistic effect of proton irradiation and strain can simulate the actual space application conditions. This result indicated that slight strain in the space environment reduced the damage caused by proton irradiation, which is beneficial to the application of the PI fiber in space.

### 3.2 Evolution of free radicals of the polyimide fibers after being irradiated

#### 3.2.1 150 keV proton irradiation

**Fig. 5:**
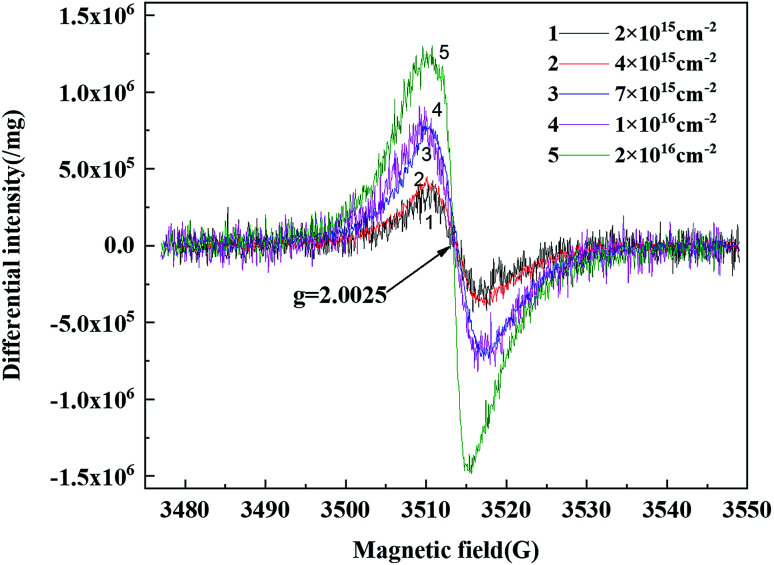
EPR spectra of 150 keV proton-irradiated polyimide fibers with different fluences.

Fig. 5 shows the electron paramagnetic resonance spectrum of the 150 keV proton-irradiated polyimide fiber. It can be seen that the *g* value of all the free radicals was 2.0025, which was independent of the irradiated fluences. According to the previous research, there are two types of free radical with this *g* value. One is a pyrolytic carbon radical^22,23^ and the other is a hydrocarbon-based superoxide radical. Generally, the hydrocarbon-based superoxide radical is only generated under environments with oxygen. Therefore, the free radical here was a pyrolytic carbon radical because the test proceeded in a vacuum environment.^23^ Furthermore, the free radical content increased with the increase in the irradiated fluences. Those results were similar to the PI films. However, the content of the free radical was far lower than for the PI film under the same conditions.^8^ Sun's research showed that the recombination of free radicals mainly occurs on the surface.^8,10^ For the fiber, its specific surface area was much larger than that of the film, so the free radicals generated by irradiation could be recombined and balanced rapidly, therefore resulting in a lower amount of free radicals.

**Fig. 6:**
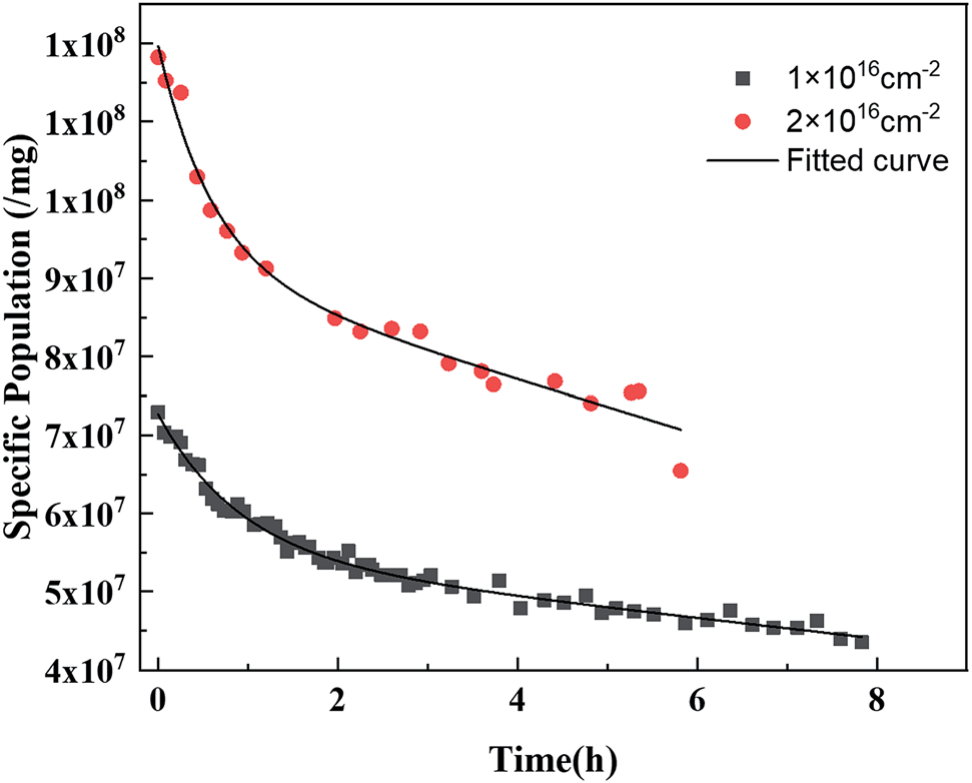
Free radical content evolution of the samples with fluences of 1 × 10^16^ cm^−2^ and 2 × 10^16^ cm^−2^ as a function of the storage time.

To clarify the recovery rate of the PI fiber after irradiation, the free radical content evolution of the samples with fluences of 1 × 10^16^ cm^−2^ and 2 × 10^16^ cm^−2^ as a function of storage time were plotted and the results are shown in Fig. 6. The result showed that the free radical content decreased with storage time. The evolution of the free radical content followed a sum of the linear and exponential modes, as shown in eqn (2):2
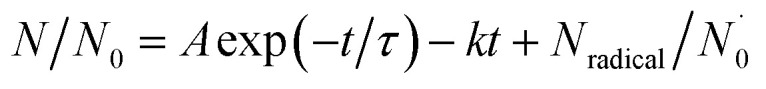
where *N* is the experimented specific free-radical population (g^−1^); *N*_0_ is the free-radical population measured immediately after irradiation (g^−1^); *A* is a constant related to the annealable free-radical population, induced by the free-radical recombination; *τ* is the characteristic time constant (h), which is related to the recovery rate of the annealable free radicals induced by the recombination process; *k* is a time constant (h^−1^) related to the effects of the surface reaction; and *N*_residual_ is the residual free-radical population (g^−1^) under the testing condition.

Table 4 shows the fitting values of *τ* and *k* of the proton-irradiated polyimide fiber during aging. The *τ* of the sample with a higher proton fluence was lower, where *τ* is the characteristic time constant of the free radical recombination, which is related to the molecular structure. In higher proton fluence, the degradation of the molecular chain caused by irradiation decreased the free volume of the PI molecules, which resulted in accelerated radical recombination, that is, *τ* decreased. The *k* of the sample with higher proton fluence was higher, which was also an indicator of the acceleration of the radical recombination. *k* is the time constant related to the effects of atmosphere. As mentioned above, the recombination of the free radicals occurred on the surface.^8,10^ With higher proton fluence, the surface roughening caused by irradiation increased the recombination area, and therefore accelerated the radical recombination.

**Table 4:** Fitting values of *τ* and *k* of the proton-irradiated polyimide during aging

Fluence (cm^−2^)	*τ* (h)	*k* (h^−1^)
1 × 10^16^	0.92	0.0180
2 × 10^16^	0.61	0.0299

The free radicals reflected the effect of radiation on the molecular structure of the polyimide fiber. The above results indicated that proton irradiation caused a large number of free radicals inside the polyimide fiber, and therefore led to the damage of its molecular structure. This further resulted in a decrease in the mechanical properties, which was consistent with the tensile test result.

#### 3.2.2 Synergistic effect of proton irradiation and strain

**Fig. 7:**
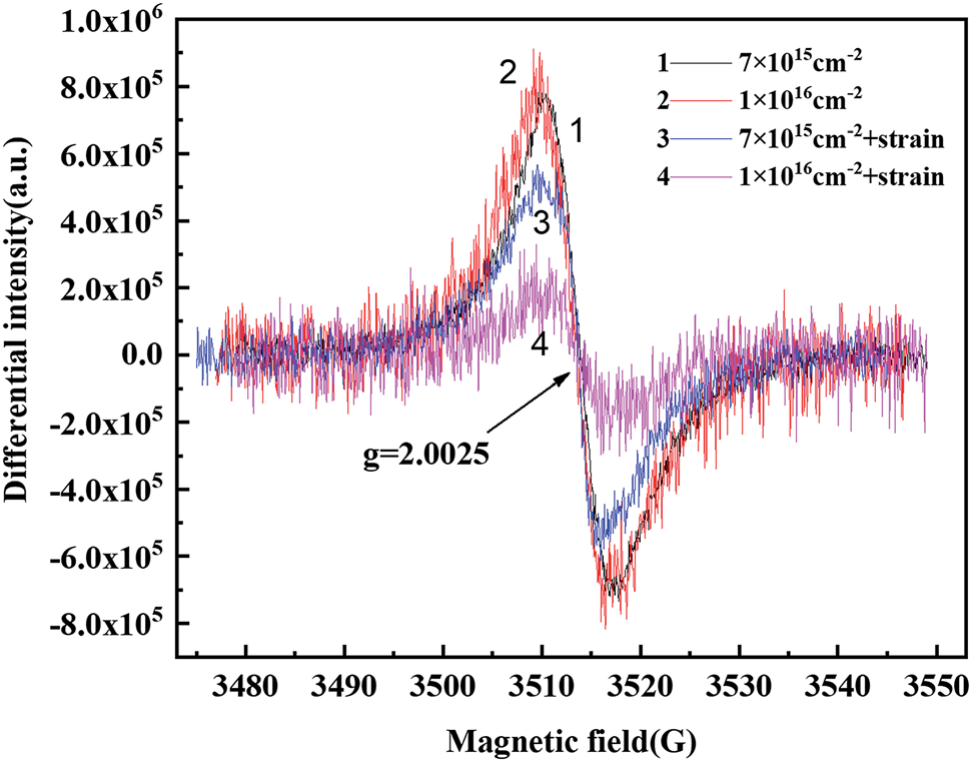
EPR spectrum of the polyimide fiber after the combination of proton irradiation and strain.

Fig. 7 shows the EPR spectrum of the polyimide fiber after the combination of proton irradiation and strain. The type of free radical was still *g* = 2.0025 after irradiation. Thus, the free radical here should be a pyrolytic carbon free radical due to the vacuum environment.

The results also showed that the free radical content for the combination of proton irradiation and strain samples was less than that in the single irradiated sample. This might be due to the increased free volumes of the polyimide molecular chain that caused by the strain, which made the radical recovery faster. In addition, the free radical content of the sample with the combination of proton irradiation and strain and with the fluence of 7 × 10^15^ cm^−2^ was higher than that with 1 × 10^16^ cm^−2^. This might because, at the same irradiation flux, the larger the irradiation fluence, the longer the time it took, coupled with a faster recovery rate of the free radical under the strain, thus resulting in a lower free radical content.

At the same time, due to the lower amount of free radicals generated after the strain application, they couldn't even be detected for a period of time after the end of irradiation. Therefore, the annealing kinetics test for the free radicals after the application of strain could not be performed here.

### 3.3 Effect of proton radiation on the chemical structure of the polyimide fiber

The composition ratio of each element in the polyimide fiber after 150 keV proton irradiation was analyzed, and the values are listed in Table 5. After proton irradiation, the proportion of each element changed, whereby the ratio of the element C increased, while the ratio of N and O decreased. The carbonization of the surface caused a brittleness of the polyimide fiber, and therefore led to reductions in the tensile strength and elongation at break, which supported the results for the mechanical properties.

**Table 5:** Composition ratio of each element in the polyimide fiber after 150 keV proton irradiation

Energy	Fluence (cm^−2^)	C%	O%	N%
As-received	—	79.92	15.15	4.93
150 keV	2 × 10^15^	84.03	11.01	4.96
	7 × 10^15^	84.82	11.46	3.72
	1 × 10^16^	84.23	12.36	3.41

There were four kinds of chemical bonds to C and their corresponding ratios are listed in Table 6. The analysis showed that the C–C content ratio increased after irradiation, while the content ratios of the other bonds decreased. Among them, the content ratio of C–N decreased the most, followed by C–O, and the least decrease was for C=O. This result showed that the flexible structure, such as C–O bonds, was damaged by irradiation, while the rigid structure, such as benzene rings (including C–C bonds), was not reduced. Therefore, the effect of proton irradiation on the modulus of the PI fiber was very small, which supported the results for the mechanical properties.

**Table 6:** Chemical bonds to C and their corresponding ratios before and after 150 keV proton irradiation

Energy	Fluence (cm^−2^)	Chemical bond	Binding energy	Ratio (%)
As-received	—	C–C	284.5	45.0
		C–N	285.4	28.2
		C–O	286.3	19.8
		C=O	288.2	7.0
150 keV	2 × 10^15^	C–C	284.6	73.9
		C–N	285.4	12.0
		C–O	286.4	11.3
		C=O	288.0	2.8
150 keV	7 × 10^15^	C–C	284.6	74.4
		C–N	285.7	14.1
		C–O	286.5	6.9
		C=O	288.1	4.6
150 keV	1 × 10^16^	C–C	284.5	87.3
		C–N	285.5	4.3
		C–O	286.2	5.1
		C=O	288.1	3.3

Table 7 shows the two kinds of chemical bonds to O and their corresponding ratios. The results showed that the ratio of C=O increased, while those of C–O decreased. This indicated that the reduction of C–O was more significant than that of C=O, which was consistent with the results for C.

**Table 7:** Chemical bonds to O and their corresponding ratios before and after 150 keV proton irradiation

Energy	Fluence (cm^−2^)	Chemical bond	Binding energy	Ratio (%)
As-received	—	C–O	531.8	69.2
		C=O	533.1	30.8
150 keV	2 × 10^15^	C–O	531.6	59.8
		C=O	533.0	40.2
150 keV	7 × 10^15^	C–O	531.8	62.0
		C=O	533.1	38.0
150 keV	1 × 10^16^	C–O	531.7	60.7
		C=O	533.0	39.3

There was only one chemical state, C–N, for N atoms before and after irradiation, while the ratio of N was reduced. This indicated that a denitrification reaction occurred on the surface of the sample during proton irradiation, which caused the decrease in the content of C–N and N atoms (as shown in Table 6). The N atoms might have escaped from the surface in the form of gas or small volatile molecules.

**Fig. 8:**
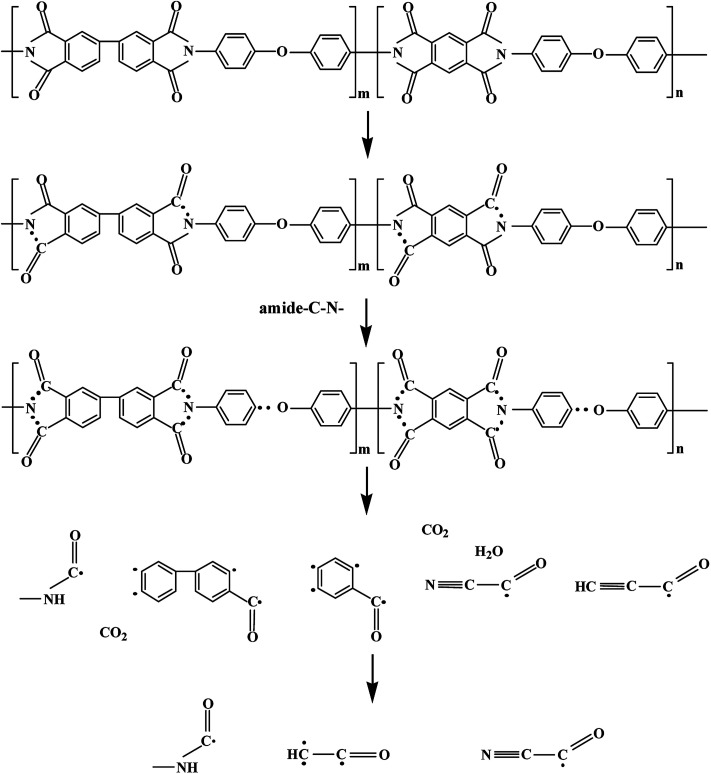
Formation mechanism of free radicals after 150 keV proton irradiation.

According to Sun,^24^ the irradiation damage process of the molecular structure of irradiated polyimides can be obtained by XPS and EPR, as shown in Fig. 8. Under proton irradiation, the internal molecular units of polyimide fibers broke and free radicals were generated. Due to the low bond energy, a large number of C–N bonds were fractured at the initial stage of proton irradiation. With the increase in proton fluence, C=O and C–O bonds were also fractured, and then a large number of free radicals were formed. With the further increase in proton fluence, a large number of small molecules were generated, such as CO_2_, H_2_O, and N_2_. The small molecule by-products diffused to the surface of the material, and overflowed from the surface to the vacuum environment, resulting in the phenomenon of material outgassing. This result was very similar with that for PI films.^24^

## 4. Conclusions

According to the above test results and analysis, the following conclusions were obtained:

(1) Proton irradiation could significantly reduce the tensile strength and elongation at break of the polyimide fiber, while slightly decreased the modulus. The synergetic effect of proton irradiation and strain weakened the reduction of the mechanical properties caused by single proton irradiation, because the strain promoted the reorganization and alignment of the molecular chains during the irradiation.

(2) After 150 keV proton irradiation, pyrolytic carbon free radicals were generated, and the number of free radicals increased with the increasing irradiation fluence, while they gradually decreased with the storage time. The decrease in the free radicals followed a sum of an exponential and linear mode. The synergetic effect of proton irradiation and strain did not change the type of free radicals formed in the polyimide fiber, while it lowered their content.

(3) According to XPS analysis, the 150 keV proton-irradiated polyimide fiber underwent complex denitrification and deoxygenation reactions, and carbon enrichment appeared on the surface of the material. The proportion of the C–N content decreased the most, followed by C–O, and then C=O, which decreased the least.

The results of this study can provide certain guidances for the application of polyimide fibers in space. However, further detailed research is needed on the mechanism of the above changes.

## Conflicts of interest

There are no conflicts to declare.

## Supplementary Material
